# Comparative study of the degradation of the 2,4-dichlorophenoxyacetic acid herbicide using zinc oxide and zinc oxide/graphene oxide photocatalysts: efficiency, stability, toxicity, kinetics and mechanism insights

**DOI:** 10.1007/s11356-026-38007-0

**Published:** 2026-07-09

**Authors:** Ana Elizabeth Rodrigues de Freitas, Flaviano Henrique de Sousa Medeiros, João Pedro Gonçalves de Souza Soares, Marcos Gomes Ghislandi, Melissa Gurgel Adeodato Vieira, Vivian Stumpf Madeira, Mauricio Alves da Motta Sobrinho

**Affiliations:** 1 Department of Chemical Engineering (DEQ), Federal University of Pernambuco (UFPE), Recife, Pernambuco 50670-901 Brazil; 2 Department of Chemical Engineering (DEQ), Federal University of Paraíba (UFPB), João Pessoa, Paraíba 58051-900 Brazil; 3 Engineering Campus (UACSA), Federal Rural University of Pernambuco (UFRPE), Cabo de Santo Agostinho, Pernambuco 54518-430 Brazil; 4 School of Chemical Engineering (FEQ), University of Campinas (UNICAMP), Campinas, São Paulo, 13083-852 Brazil

**Keywords:** Photocatalysis, Graphene oxide, Zinc oxide, Herbicide, 2,4-D, Toxicity, *Lactuca sativa*

## Abstract

This study investigated the degradation of the herbicide 2,4-dichlorophenoxyacetic acid (2,4-D) in aqueous solution through heterogeneous photocatalysis under simulated solar irradiation. For this purpose, zinc oxide (ZnO), graphene oxide (GO), and a ZnO/GO composite were synthesized and characterized using Fourier-transform infrared spectroscopy (FTIR), X-ray diffraction (XRD), dynamic light scattering (DLS), scanning electron microscopy (SEM), energy-dispersive spectroscopy (EDS), diffuse reflectance spectroscopy (DRS), and photoluminescence (PL) analyses. Compared with pure ZnO, the ZnO/GO composite exhibited enhanced structural, morphological, and optical properties owing to the incorporation of GO. These improvements included a reduction in particle size (from 15.565 to 9.528 µm), a more homogeneous dispersion of ZnO particles on the GO sheets, enhanced visible-light absorption, and more efficient separation of photogenerated charge carriers. As a result, the composite demonstrated superior photocatalytic performance, achieving 96.6% degradation of a 2,4-D aqueous solution (10 mg L^−1^) within 150 min using a photocatalyst dosage of 0.25 g L^−1^. Under the same experimental conditions, pure ZnO achieved a degradation efficiency of 88.5%. Furthermore, the ZnO/GO composite showed a greater mineralization capacity, reaching 84.82% total organic carbon (TOC) removal, whereas ZnO achieved 73.60% TOC removal. The chemical stability of both photocatalysts was confirmed over three consecutive reuse cycles. In addition, the treated solutions obtained after photocatalytic degradation using either ZnO or ZnO/GO did not exhibit phytotoxic effects toward Deva lettuce seeds (*Lactuca sativa*). However, the relative growth index (RGI) obtained for the solution treated with the ZnO/GO composite was approximately 3.2 times higher than that observed for ZnO. Overall, the ZnO/GO composite outperformed pure ZnO in all evaluated aspects, demonstrating that the incorporation of GO enhances the intrinsic properties of ZnO and contributes to the development of a more efficient photocatalytic material for the degradation of emerging contaminants in water.

## Introduction

The continuous growth of the global population and the resulting increase in food demand have driven the widespread and intensive use of herbicides in modern agriculture (Pereira et al. 2023; Zhang et al. 2024). Among these compounds, 2,4-dichlorophenoxyacetic acid (2,4-D) is one of the most extensively used herbicides for the control of broadleaf weeds, playing a key role in enhancing agricultural productivity. However, its extensive application has led to its increasing detection in aquatic environments, raising concerns regarding its potential environmental and ecological impacts (Cornish and Sweetman 2023; Martins et al. 2024).

2,4-D is commonly applied in wheat, corn, rice, cereal, and sugarcane crops to suppress the growth of broadleaf weeds. When applied at appropriate concentrations, this herbicide promotes the activation of auxin-related genes, particularly those involved in the biosynthesis of ethylene and abscisic acid. The overproduction of these phytohormones disrupts essential physiological processes, including cell division and growth regulation, accelerating leaf senescence and ultimately leading to the inhibition and death of susceptible weed species (Martins et al. 2024).

Despite its importance in agricultural production, 2,4-D exhibits relatively low soil adsorption and high water solubility, characteristics that favor its mobility in the environment. Consequently, the herbicide can leach into surface and groundwater systems and may eventually reach drinking water reservoirs (Lan et al. 2024; Zhang et al. 2024; Chen et al. 2024). Concerns regarding the occurrence of 2,4-D in aquatic environments are primarily associated with its adverse effects on non-target organisms. In this regard, an increasing number of studies have reported the toxicological and ecological consequences of prolonged exposure to this compound in different species (Gaaied et al. 2019; Tichati et al. 2020, 2021; Wang et al. 2022; Silva et al. 2024).

The low biodegradability and recalcitrant nature of 2,4-D hinder its removal by conventional biological treatments and physicochemical processes, such as coagulation, flocculation, sedimentation, filtration, and disinfection. In addition, concerns related to its chemical stability, the possible formation of byproducts that may be more toxic than the parent compound, and the frequent need for post-treatment processes represent important limitations of these conventional approaches (Lan et al. 2024). Given the toxicological risks associated with 2,4-D and its environmental persistence, the development of effective treatment technologies for its removal from aquatic systems is of considerable importance.

Among the technologies currently employed for the treatment of water contaminated with 2,4-D, adsorption and advanced oxidation processes (AOPs) have received significant attention. Examples of AOPs include ozonation, Fenton and photo-Fenton reactions, UV/persulfate, UV/hydrogen peroxide, and photocatalytic processes (Singh et al. 2021; Khan et al. 2021). While adsorption promotes the transfer of the contaminant from the aqueous phase to the adsorbent surface without chemical transformation, AOPs are capable of degrading recalcitrant organic pollutants and, in many cases, achieving their complete mineralization into carbon dioxide and water (Yu and Huang 2023).

Among the various AOPs, photocatalysis has emerged as a promising and environmentally friendly technology for the degradation of organic contaminants, enabling the generation of less harmful products while allowing catalyst recovery and reuse (Yu and Huang 2023). This process has been widely investigated for the removal of 2,4-D from aqueous solutions (Jin et al. 2023; Lan et al. 2024; Gopal et al. 2024; Fávaro et al. 2024).

A key challenge in photocatalytic applications is the development of materials with enhanced photocatalytic performance, particularly those capable of improving charge-carrier separation and extending light absorption into the visible region. The importance of these properties has been demonstrated in several ZnO-based photocatalytic systems. For instance, Sayed et al. (2021) reported that the simultaneous enhancement of visible-light absorption and charge-carrier separation significantly improved the photocatalytic performance of Mn,C-codoped ZnO hollow structures. These findings highlight that strategies aimed at reducing electron–hole recombination and increasing light utilization are essential for the development of highly efficient photocatalysts. Among the various approaches explored, one of the most effective is the combination of two or more semiconducting materials to form composite systems, which can promote synergistic effects and improve the optical and electronic properties of the photocatalyst (Jia et al. 2023; Yin et al. 2023).

Zinc oxide (ZnO) is one of the most extensively studied semiconductor photocatalysts due to its attractive physicochemical properties, including high chemical, thermal, and mechanical stability, strong room-temperature luminescence, and low biotoxicity (Atta-Eyison et al. 2021; Raha and Ahmaruzzaman 2022). Nevertheless, ZnO presents some intrinsic limitations that restrict its photocatalytic efficiency. Its relatively wide band-gap energy (3.37 eV) limits light absorption predominantly to the ultraviolet region, reducing its activity under visible-light irradiation (Masar et al. 2023). Moreover, ZnO is characterized by a high recombination rate of photogenerated electron–hole pairs and a tendency to form relatively large particles, both of which negatively affect its photocatalytic performance (Gindose et al. 2024, 2025; Yang et al. 2026).

To overcome the intrinsic limitations of ZnO, one of the most effective strategies is to combine it with other materials to develop photocatalysts with improved optical, electronic, and photocatalytic properties (Gindose et al. 2024, 2025). Among the available alternatives, graphene oxide (GO) has emerged as a particularly promising material due to its unique physicochemical characteristics. GO possesses a high specific surface area of approximately 2630 m^2^ g^−1^ and excellent electrical conductivity, which facilitate electron transport across the catalyst surface and, consequently, enhance photocatalytic performance (Neolaka et al. 2020; Al Kausor and Chakrabortty 2021). In addition, graphene-based materials can act as electron acceptors owing to their two-dimensional structure, promoting more efficient separation of photogenerated electron–hole pairs and suppressing charge-carrier recombination (Ahmed and Mohamed 2023). These materials may also improve light absorption in the visible region, further contributing to enhanced photocatalytic activity (Mylsamy et al. 2025).

Recent studies have demonstrated that coupling ZnO with conductive or semiconducting materials is an effective strategy for improving charge-carrier separation and enhancing photocatalytic performance. For example, Liu et al. (2025a) reported that the construction of a ZnO-based heterojunction significantly improved the separation and migration of photogenerated charge carriers, resulting in enhanced photocatalytic degradation and mineralization of organic pollutants. Likewise, the development of hybrid photocatalytic systems incorporating carbon-based materials has attracted considerable attention due to their ability to facilitate electron transport, suppress charge recombination, and extend the lifetime of reactive species involved in photocatalytic reactions. These findings reinforce the potential of ZnO-based composite materials as efficient photocatalysts for environmental remediation applications.

Given the environmental concerns associated with 2,4-D contamination and the need for efficient treatment technologies, this study focused on the synthesis and evaluation of zinc oxide (ZnO) and zinc oxide/graphene oxide (ZnO/GO) photocatalysts for the photocatalytic degradation of this herbicide. The main objective was to compare the performance of these materials in terms of degradation efficiency, chemical stability, reaction kinetics, toxicity, and photocatalytic mechanism, thereby providing a comprehensive assessment of the benefits associated with the incorporation of GO into the ZnO structure. By advancing the understanding of ZnO/GO-based photocatalytic systems for herbicide removal, this study contributes to the development of more effective strategies for mitigating the environmental impacts of 2,4-D contamination in aquatic environments.

## Materials and methods

### Materials and chemicals

Zinc nitrate hexahydrate (Zn(NO_3_)_2_.6H2O) (purity ≥ 96% P.A., Dinâmica Química, Brazil), citric acid (C_6_H_8_O_7_) (purity ≥ 99 P.A., Synth, Brazil), ethylene glycol (C_2_H_6_O_2_) (purity ≥ 99% P.A., Neon, Brazil), graphite powder (99% P.A., Synth, Brazil), sulfuric acid (H_2_SO_4_ 95% P.A., Anidrol, Brazil), potassium permanganate (KMnO_4_ 99% P.A., Química Moderna, Brazil), hydrogen peroxide (H_2_O_2_, 35% w/w, Dinâmica Química, Brazil) and hydrochloric acid (HCl 37% P.A., Anidrol, Brazil) were used in the synthesis of the catalysts. The herbicide evaluated in the photodegradation assays was 2,4-dichlorophenoxyacetic acid (2,4-D, purity ≥ 95%, Sigma-Aldrich). Silver nitrate (AgNO_3_) (P.A., PLAT-LAB, Brazil), ascorbic acid (C_6_H_8_O_6_) (purity ≥ 99%, P.A., INLAB, Brazil), ammonium oxalate ((NH_4_)_2_C_2_O_4_.H_2_O) (purity ≥ 99%, P.A., Dinâmica Química, Brazil), isopropyl alcohol (C_3_H_8_O) (purity ≥ 99.5%, Neon, Brazil), phosphoric acid (H_3_PO_4_) (purity ≥ 85% P.A., Fmaia, Brazil), and acetonitrile (CH_3_CN) (gradient grade for liquid chromatography, LiChrosolv® Reag. Ph Eur., purity ≥ 99.9%, Merck) were used in the scavenger experiments. All solutions were prepared using distilled water, and all reagents were used as received without any further purification or treatment.

### Synthesis of pure photocatalysts

Graphene oxide (GO) was synthesized using an adapted version of the modified Hummers method (Hummers and Offeman 1958), following the methodology described by Araujo et al. (2018). The reaction mixture was maintained at 35 ± 5 °C for 6 h and subsequently terminated by the addition of H_2_O_2_. The resulting GO was washed successively with distilled water and a 5% (v/v) HCl solution until neutral pH was achieved. The material was then exfoliated in an ultrasonic bath for 4 h.

ZnO was synthesized using the Pechini sol–gel method, according to the procedure reported by Brasileiro et al. (2023). The obtained powder was calcined in a muffle furnace at 500 °C for 2 h using a heating rate of 10 °C min^−1^. Subsequently, the material was washed until neutral pH was reached to remove residual species not incorporated into the oxide structure and then dried in an oven at 110 °C for 24 h.

### Synthesis of ZnO/GO composite

The ZnO/GO composite photocatalyst was synthesized using a mixing-assisted ultrasonication method, following the procedure described by Sher et al. (2021), with a graphene oxide content of 10 wt%. Initially, ZnO and GO were separately dispersed in distilled water under mechanical stirring for 30 min. The resulting suspensions were then combined and stirred for an additional 30 min to promote homogeneous mixing. Subsequently, the mixture was subjected to ultrasonic treatment for 1 h to enhance the interaction between the ZnO particles and GO sheets. After sonication, the suspension was dried in an oven at 45 °C for 48 h. The dried material was then ground and sieved through a 200-mesh ABNT sieve to obtain a homogeneous composite powder.

### Characterization

The synthesized photocatalysts were characterized using Fourier-transform infrared spectroscopy (FTIR), X-ray diffraction (XRD), dynamic light scattering (DLS), scanning electron microscopy (SEM), energy-dispersive spectroscopy (EDS), diffuse reflectance spectroscopy (DRS), and photoluminescence (PL) analyses. The surface functional groups of the photocatalysts were identified by FTIR using a Shimadzu IRPrestige-21 spectrophotometer. The spectra were recorded in the wavenumber range of 4000–400 cm^−1^, with a spectral resolution of 4 cm^−1^.

The crystalline structure and phase purity of the materials were investigated by XRD using a Shimadzu LabX XRD-6000 diffractometer operating with Cu Kα radiation at 30 kV and 30 mA, corresponding to a power of 2 kVA. Diffraction patterns were collected over a 2θ range of 5–80°, using a step size of 0.02° and a scanning rate of 0.5° min^−1^.

The crystallite diameter (D_c_) was estimated using the Scherrer equation (Eq. 1) (Liu et al. 2025b):

1 $${\mathrm D}_{\mathrm c}=\frac{\kappa\lambda}{\beta\cos\left(\mathrm\theta\right)}$$

where K is the Scherrer constant (K = 0.9), λ is the wavelength of copper Kα radiation (λ = 1.54184 Å), θ is the Bragg diffraction angle, and β is the full width at half height (FWHM) of the diffraction peak.

The particle size (P_S_) of the synthesized materials was determined by dynamic light scattering (DLS) using a Zetasizer Nano ZS90 instrument (Malvern Panalytical, USA). The refractive indices adopted for ZnO, GO, and ZnO/GO were 2.0, 2.42, and 2.0, respectively (Heideman et al. 1995; Farivar et al. 2021). Measurements were performed in distilled water using suspensions containing 0.01 wt% of each sample after dispersion in an ultrasonic bath for 20 min.

The morphology of the materials was examined by scanning electron microscopy (SEM) using a VEGA 3 microscope (Tescan). The elemental composition of the samples was determined by energy-dispersive X-ray spectroscopy (EDS), using a detector coupled to the SEM system.

The optical properties of the photocatalysts were investigated by UV–Vis diffuse reflectance spectroscopy (DRS) in the wavelength range of 200–800 nm using a Shimadzu UV-2600 spectrophotometer.

Photoluminescence (PL) spectroscopy was employed to evaluate the recombination behavior of photogenerated charge carriers. The PL spectra were recorded using a Fluorolog-3 spectrofluorometer (Horiba) with an excitation wavelength of 340 nm.

### Characterization of the solutions

The amount of organic matter present in the initial 2,4-D solution and in the treated solutions obtained after the heterogeneous photocatalytic process was quantified using a total organic carbon analyzer (TOC-L, Shimadzu).

### Photocatalytic activity

The synthesized photocatalysts were evaluated through photocatalytic degradation experiments to assess their ability to remove 2,4-dichlorophenoxyacetic acid (2,4-D) from aqueous solution. The experiments were carried out in a glass reactor placed inside a ventilated enclosure to maintain a constant operating temperature. All tests were conducted in batch mode under continuous mechanical stirring at 300 rpm and simulated solar irradiation. A 300 W high-pressure tungsten lamp (Ultra-Vitalux, Osram) was used as the light source, emitting radiation within the wavelength range of 280–780 nm and positioned at a fixed distance of 10 cm from the solution surface.

Each experiment was performed in a 400 mL glass reactor containing 250 mL of a 2,4-D solution (10 mg L^−1^) and 0.25 g L^−1^ of catalyst, of photocatalyst, which remained suspended throughout the reaction. The herbicide solution was used at its natural pH (5.0 ± 0.5). Prior to irradiation, the suspensions were kept in the dark for 30 min to establish adsorption–desorption equilibrium between the photocatalyst surface and the pollutant molecules. Subsequently, the samples were exposed to simulated solar irradiation for 150 min.

During the photocatalytic reaction, 2.5 mL aliquots were collected at predetermined time intervals, filtered through a 0.22 μm PVDF syringe filter, and analyzed. The concentration of 2,4-D was monitored by UV–Vis spectrophotometry (UV-1280, Shimadzu) at a wavelength of 283 nm. The degradation efficiency of 2,4-D was calculated according to Eq. 2, where C_0_ represents the initial concentration of 2,4-D and C(*t*) corresponds to the concentration of the herbicide at a given reaction time *t* (mg L^−1^).

2 $$\mathrm{Degradation}\;\mathrm{efficiency}=\left(1-\frac{\mathrm{C}}{{\mathrm{C}}_0}\right).100$$

### Chemical stability assessment

The chemical stability of the photocatalysts was evaluated through reusability tests conducted over three consecutive photocatalytic cycles. After each cycle, the catalyst was recovered by filtration, washed with distilled water, dried in an oven at 45 °C for 24 h, and subsequently reused under the same experimental conditions.

### Ecotoxicity tests

The toxicity of both the initial 2,4-D solution and the treated solutions obtained after the photocatalytic process was assessed through acute ecotoxicity tests using Deva lettuce (*Lactuca sativa*) seeds as bioindicators. For each assay, 20 seeds were placed on filter paper inside a Petri dish and moistened with 4 mL of the corresponding sample solution. Distilled water and a 3% boric acid solution were used as negative and positive controls, respectively. All experiments were performed in quintuplicate.

The Petri dishes were incubated in the absence of light for 120 h at 22 ± 2 °C. After the incubation period, the number of germinated seeds and the root lengths were recorded. The toxicity of the samples was evaluated using the germination index (GI) (Eq. 3) and the relative growth index (RGI) (Eq. 4), according to the methodology proposed by Soares et al. (2013).

3 $$\mathrm{GI}\;\left(\mathrm{\%}\right)=\left(\frac{{\mathrm{S}}_\mathrm{G}}{{\mathrm{S}}_\mathrm{T}}\right).100$$

4 $$\mathrm{R}\mathrm{G}\mathrm{I}=\frac{{\mathrm{C}\mathrm{R}}_{\mathrm{i}}}{{\mathrm{C}\mathrm{R}}_{\mathrm{n}}}$$

where S_G_ and S_T_ represent the number of germinated and total seeds, respectively, CR_i_ is the root growth in sample i, and CR_n_ is the growth in the negative control.

### Detection of reactive species

Reactive-species scavenging experiments were conducted to evaluate the contribution of the main active species involved in the photocatalytic degradation of 2,4-D and, consequently, to propose a plausible reaction mechanism. For this purpose, isopropyl alcohol (IPA), ammonium oxalate (AO), silver nitrate (AgNO_3_), and ascorbic acid (AA) were employed as scavengers for hydroxyl radicals (^•^OH), photogenerated holes (h^+^), electrons (e^−^), and superoxide radicals (O_2_^•⁻^), respectively. Each scavenger was evaluated individually and added to the reaction solution prior to the photocatalytic experiments. In all assays, the scavenger concentration was fixed at 0.01 M.

The samples collected after the photocatalytic experiments were analyzed by high-performance liquid chromatography (HPLC) using a Shimadzu 10-A HPLC system equipped with an SPD-M10AVP diode-array detector (DAD). The mobile phase consisted of a mixture of 65% acetonitrile, 34.9% distilled water, and 0.1% phosphoric acid, delivered at a flow rate of 1 mL min^−1^. Chromatographic separation was performed using an Agilent Zorbax SB-C8 column (4.6 × 150 mm) connected in series with a Shim-pack C18 column (4.6 × 250 mm).

## Results and discussion

### Identification of functional groups (FTIR)

**Fig. 1:**
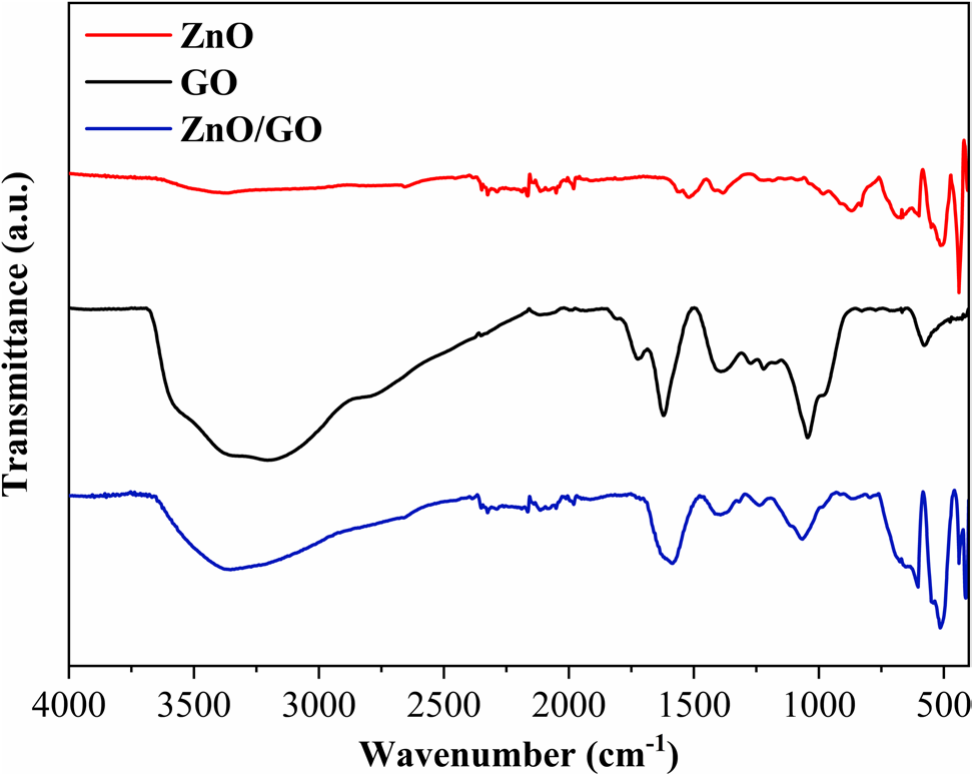
FTIR spectra of ZnO, GO and ZnO/GO composite

The FTIR spectra of pure ZnO, pure GO, and the ZnO/GO composite are displayed in Fig. 1. For the pure ZnO sample, the spectrum exhibits a flat profile in the higher wavenumber region, with only a very weak and shallow broad band between 3600–3200 cm^−1^. This faint signal is attributed to the O–H stretching vibrations of residual surface hydroxyl groups and a minimal amount of physisorbed water molecules from ambient moisture (Rhoden et al. 2021). Furthermore, the absence of a prominent peak at approximately 1620 cm^−1^ confirms that the synthesized ZnO possesses a low surface hydration state under the measurement conditions. The sharp and highly intense absorption band observed below 1000 cm^−1^ corresponds to the characteristic vibrational mode of the Zn–O bond (Benitez-Salazar et al. 2021; Fatima et al. 2022), confirming the successful formation of a well-crystallized zinc oxide framework.

In contrast, the FTIR spectrum of pure GO presents intense and well-defined bands characteristic of highly oxygenated graphene sheets. The prominent, broad absorption band centered at approximately 3400 cm^−1^ is assigned to the O–H stretching vibrations of both adsorbed water molecules and the structural hydroxyl/phenolic groups attached to the graphitic lattice (Huang et al. 2024; Shaha et al. 2024). The sharp peak at ∼ 1720 cm^−1^ is assigned to the C = O stretching vibration of carbonyl and carboxyl groups located mainly at the edges of the GO sheets. The distinct band at ~ 1620 cm^−1^ is primarily attributed to the C = C skeletal stretching vibrations of the unoxidized *sp*^*2*^ graphitic domains (Neolaka et al. 2020), which may overlap with the bending vibration mode (*δ*H-O–H) of physisorbed water. Additionally, the bands observed at ∼ 1370 cm^−1^ and ∼ 1050 cm^−1^ correspond to the C–O stretching vibrations of alkoxy and epoxy functional groups, respectively.

The ZnO/GO composite spectrum successfully combines the vibrational fingerprints of both precursors, confirming their effective integration. The composite clearly retains the Zn–O lattice bands below 1000 cm^−1^, alongside the re-emergence of the characteristic GO bands, such as the C = C aromatic skeletal vibration at ∼ 1600 cm^−1^. Interestingly, the broad O–H band centered at ∼ 3400 cm^−1^ becomes significantly more pronounced in the composite compared to pure ZnO. This enhanced density of surface hydroxyl species and bound water on the composite surface plays a crucial role in photocatalysis, as it facilitates the capture of photogenerated holes to produce highly reactive hydroxyl radicals (^•^OH), thereby boosting the degradation efficiency of the 2,4-dichlorophenoxyacetic acid herbicide.

### Analysis of crystal structure, crystallite size (XRD), and particle size (DLS)

**Fig. 2:**
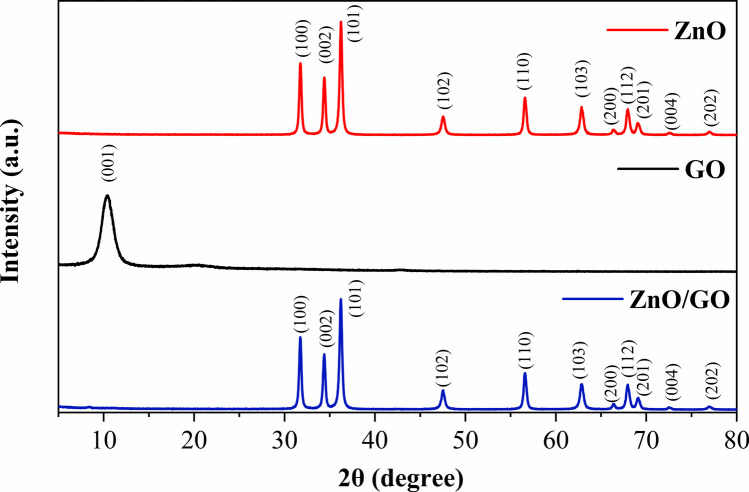
XRD patterns of ZnO, GO and ZnO/GO composite

The XRD patterns of all synthesized materials are presented in Fig. 2. The ZnO sample exhibited intense and well-defined diffraction peaks, indicating that the synthesis method and experimental conditions were effective in producing a highly crystalline material. The diffraction peaks were observed at 2θ values of 31.770°, 34.422°, 36.253°, 47.539°, 56.603°, 62.864°, 66.380°, 67.963°, 69.100°, 72.562°, and 76.955°, corresponding to the crystallographic planes (100), (002), (101), (102), (110), (103), (200), (112), (201), (004), and (202), respectively.

These diffraction peaks can be indexed to the hexagonal wurtzite structure of ZnO, in agreement with the standard reference pattern JCPDS No. 36–1451. The absence of secondary diffraction peaks further indicates the high phase purity of the synthesized ZnO and confirms the successful formation of the desired crystalline phase.

The XRD pattern of GO exhibits a characteristic diffraction peak at 2θ = 10.3°, which is assigned to the (001) crystallographic plane (Fani et al. 2022; Das et al. 2023). This peak is associated with the interlayer spacing of the graphitic sheets within the GO structure. In pristine graphite, the characteristic diffraction peak typically appears at approximately 26°. However, during the oxidation process, oxygen-containing functional groups are introduced onto the graphite layers, leading to an expansion of the interlayer distance and a consequent shift of the diffraction peak toward lower diffraction angles (Yasir et al. 2024).

**Fig. 3:**
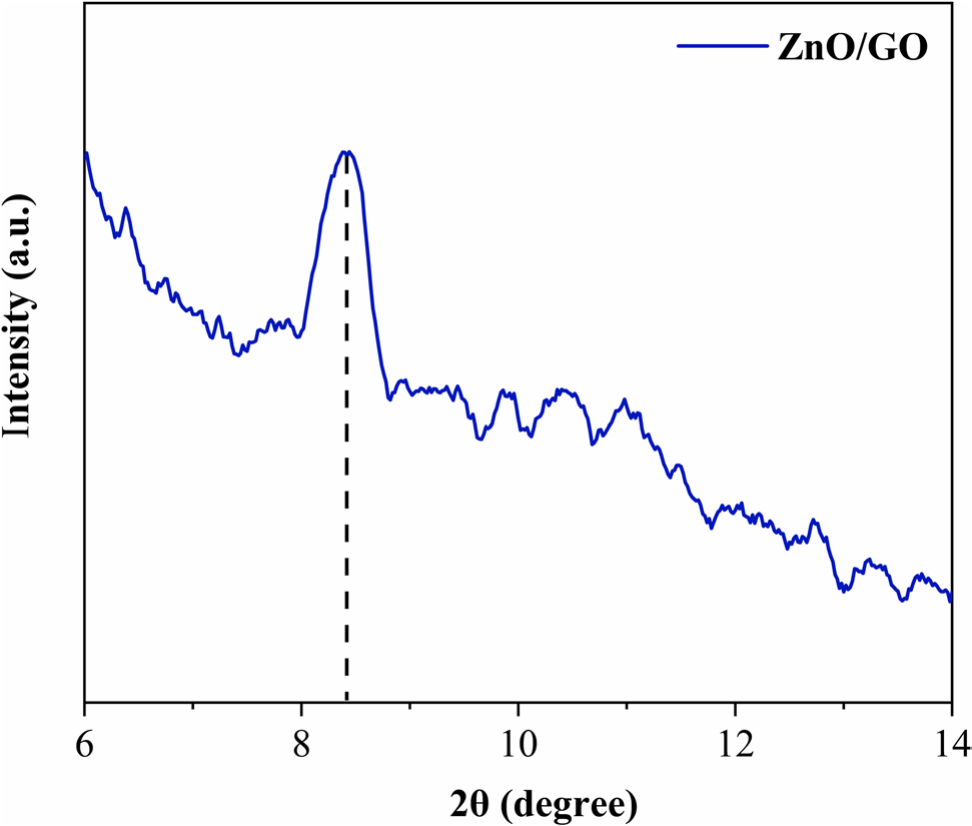
Inset of the XRD pattern showing the peak attributed to GO

The XRD pattern of the ZnO/GO composite exhibits a weak diffraction peak that is absent in the pure ZnO sample and can be attributed to the (001) plane of GO. The low intensity of this peak may be related to the relatively small amount of GO incorporated into the composite, as previously reported by Mohamed et al. (2019) and Wang et al. (2017), and/or to the intrinsically lower diffraction intensity of graphene oxide compared with ZnO (Kaur et al. 2024). To facilitate visualization, an enlarged view of the corresponding region is presented in Fig. 3, highlighting the characteristic GO diffraction peak (001) in the ZnO/GO composite.

The crystallite diameter (D_C_) estimated using the Scherrer equation and the particle size (P_S_) determined by DLS for the ZnO, GO, and ZnO/GO samples are summarized in Table 1. Crystallite size is an important parameter in photocatalytic systems, as smaller crystallites generally provide a higher density of active sites available for photocatalytic reactions (Liu et al. 2024). In addition, a reduction in crystallite size can enhance charge-carrier dynamics by shortening the migration distance of photogenerated electrons and holes to the catalyst surface, thereby decreasing the probability of electron–hole recombination and improving photocatalytic efficiency.

**Table 1:** Crystallite diameter (D_C_) and particle size (P_S_) of ZnO, GO and ZnO/GO composite

Sample	D_C_ (nm)	P_S_ (µm)
ZnO	35.39	15.565
GO	5.20	2.071
ZnO/GO	32.68	9.528

Despite their small crystallite diameters, the samples exhibited relatively large particle sizes, suggesting significant agglomeration. This behavior may be associated with the Pechini method employed for ZnO synthesis.

Nevertheless, the incorporation of GO into the composite resulted in a substantial reduction in particle size, decreasing from 15.565 to 9.528 µm, as determined by DLS measurements. This finding is particularly relevant for photocatalytic applications, as a smaller particle size generally increases the available surface area and the number of accessible active sites. Consequently, it can enhance the adsorption of pollutant molecules onto the photocatalyst surface, which constitutes a crucial initial step in the photocatalytic degradation process (Medvedev et al. 2022; Ye et al. 2023).

### Analysis of surface morphology and elemental composition (SEM/EDS)

**Fig. 4:**
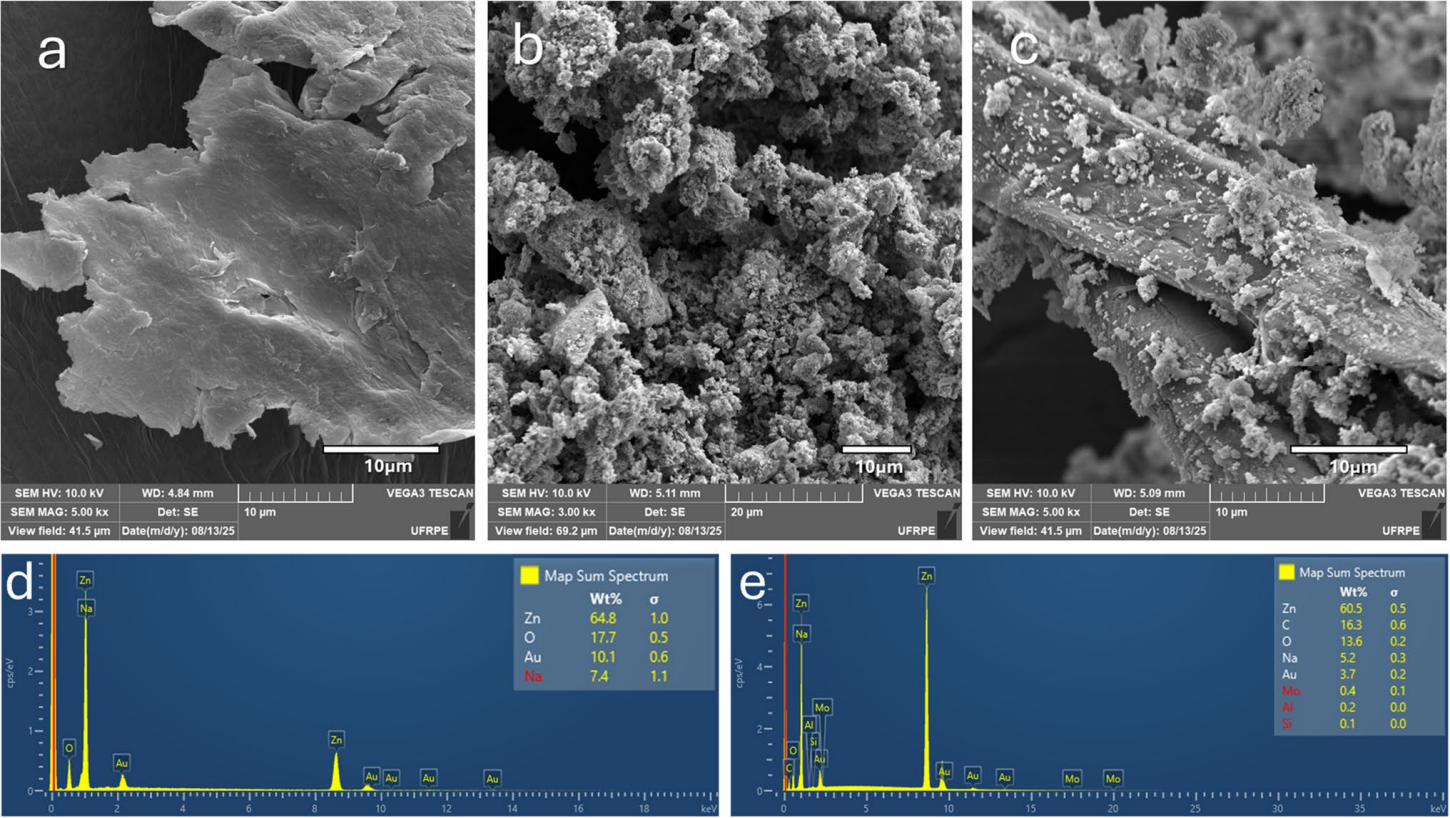
SEM micrographs of (**a**) GO, (**b**) ZnO, and (**c**) the ZnO/GO composite, together with the EDS spectra of (**d**) ZnO and (**e**) ZnO/GO

The surface morphology of the synthesized photocatalysts was investigated by scanning electron microscopy (SEM). As shown in Fig. 4a, the SEM micrograph of GO reveals its characteristic layered structure, consisting of thin and stacked sheets with irregular edges (Nascimento et al. 2022). The material also exhibits a rough and wrinkled surface morphology, which is likely associated with the structural distortions induced by the oxidation, exfoliation, and subsequent restacking processes involved in GO synthesis (Panicker et al. 2021).

The SEM micrograph of pure ZnO (Fig. 4b) reveals irregularly shaped particles with different sizes, forming micrometer-scale agglomerates. In contrast, the ZnO/GO composite (Fig. 4c) exhibits ZnO particles distributed over the surface of the stacked GO sheets, suggesting that the graphene oxide acts as a supporting substrate for ZnO deposition. This structural arrangement can increase the available surface area and promote the adsorption of reactant species, such as 2,4-D, H_2_O, and O_2_, thereby enhancing photocatalytic performance (Ahmad et al. 2021). Furthermore, the presence of GO appears to improve the dispersion of ZnO particles throughout the composite structure, in agreement with the observations reported by Fogaça et al. (2023).

The elemental composition of the samples was evaluated by EDS analysis (Fig. 4d and e). For pure ZnO, the spectrum is predominantly composed of Zn and O signals, as expected for zinc oxide structures. After the incorporation of GO, a distinct carbon signal becomes evident in the ZnO/GO spectrum, accompanied by a proportional increase in the oxygen content, which can be attributed to the oxygen-containing functional groups present in the oxidized graphitic structure of GO (Fig. 4e). Trace amounts of additional elements were also detected and are likely associated with residual species originating from the synthesis procedures. The presence of Au is attributed to the gold coating applied during sample preparation for SEM analysis.

### Analysis of optical absorption properties (DRS)

The optical absorption properties of the synthesized materials were investigated by UV–Vis diffuse reflectance spectroscopy (DRS) in the wavelength range of 220–800 nm. Diffuse reflectance spectroscopy is a valuable technique for evaluating the light-harvesting ability of photocatalytic materials and provides important information regarding their optical response and potential activity under irradiation. The optical band gap energy (E_g_) calculations were performed according to the method proposed by Wood and Tauc (1972), using the relationship described by Eq. 5.

5 $$\upalpha \mathrm{h}\upnu =\mathrm{k}{(\mathrm{h}\upnu -{\mathrm{E}}_{\mathrm{g}})}^{{~}^{\mathrm{n}}\!\left/ \!{~}_{2}\right.}$$

where α is the absorption coefficient obtained according to the Kubelka–Munk theory (Kar et al. 2019), h*ν* is the photon energy, k is an empirical proportionality constant, E_g_ is the band gap, and n is related to the type of transition.

For indirect-band-gap semiconductors, such as ZnO and GO, n = 4 (Chen et al. 2015; Yashas et al. 2021). The band gaps were estimated by extrapolating the linear region of the Tauc plot of (αh•)^n/2^ *versus* the photon energy (eV).

**Fig. 5:**
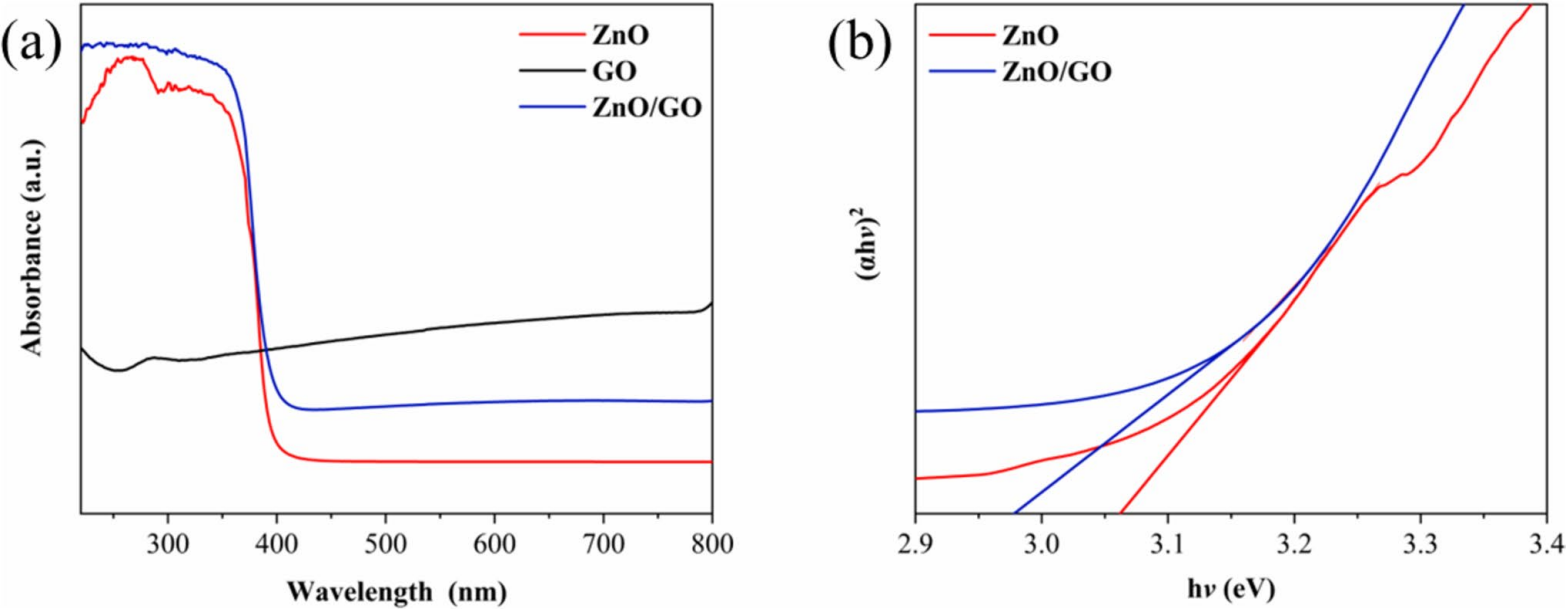
(**a**) UV–Vis absorption spectra of ZnO, GO and ZnO/GO; and (**b**) Tauc plot of ZnO and ZnO/GO

The corresponding UV–Vis absorption spectra are presented in Fig. 5a, and the optical band gap energies of the synthesized materials were estimated from the Tauc plots derived from the DRS data, as shown in Fig. 5b.

As shown in Fig. 5a, GO exhibits absorption throughout the UV–Vis region, whereas both ZnO and ZnO/GO display preferential absorption in the ultraviolet region (λ < 400 nm). However, the incorporation of GO causes a slight red shift in the absorption edge, indicating enhanced optical absorption in the visible-light region compared with pure ZnO. This shift is associated with band-gap narrowing and a more efficient utilization of incident photons, which promotes the generation of electron–hole pairs (Mylsamy et al. 2025). Similar shifts in the absorption edge following the incorporation of graphene-based materials into semiconductor structures have been reported by Chen et al. (2021), Mylsamy et al. (2025), and Wu et al. (2022).

The band-gap energy of the ZnO/GO sample, estimated from the Tauc plot (Fig. 5b), was 2.98 eV, which is lower than that of pure ZnO (3.06 eV). These results are consistent with the absorption spectra presented in Fig. 5a and confirm a red shift in the optical absorption edge from 413 nm for ZnO to 421 nm for ZnO/GO.

With a decrease in the band gap, absorption extends to longer wavelengths, improving the photocatalytic activity of the materials under visible light (Fatima et al. 2022; Warshagha and Muneer 2022). The positions of the valence band (VB) and conduction band (CB) of ZnO were estimated using the empirical equations presented in Eqs. (6) and (7) (Palanivel et al. 2019; Ren et al. 2023).

6 $${\mathrm{E}}_{\mathrm{V}\mathrm{B}}=0.5{\mathrm{E}}_{\mathrm{g}}+\upchi -{\mathrm{E}}_{\mathrm{e}}$$

7 $${\mathrm{E}}_{\mathrm{C}\mathrm{B}}={\mathrm{E}}_{\mathrm{V}\mathrm{B}}-{\mathrm{E}}_{\mathrm{g}}$$

where E_VB_ is the valence band energy, χ is the absolute electronegativity of the semiconductor, E_g_ is the band gap energy, E_e_ is the free electron energy on the hydrogen scale (4.5 eV), and E_CB_ is the conduction band energy.

For ZnO, the absolute electronegativity value χ is 5.79 eV (Xu and Schoonen 2000). Based on Eqs. (6) and (7), the calculated values of E_VB_ and E_CB_ were 2.82 and −0.24 eV, respectively.

### Investigation of photogenerated charge separation (PL)

**Fig. 6:**
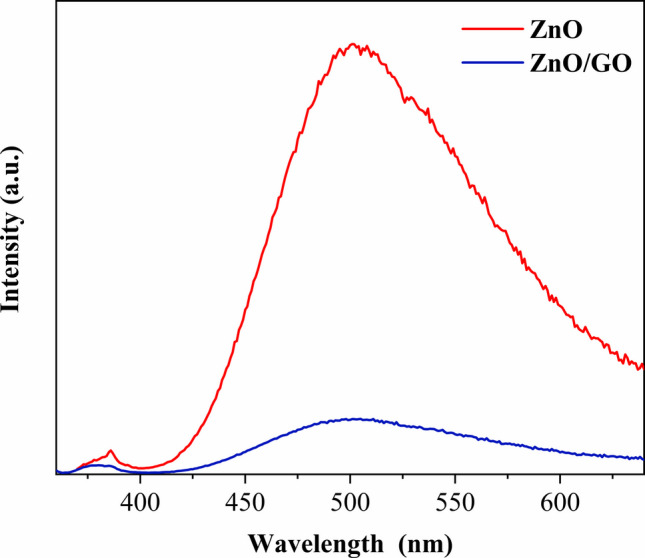
PL spectra of ZnO and ZnO/GO

Photoluminescence (PL) spectroscopy was employed to investigate the separation of photogenerated charge carriers and the transfer of electrons from ZnO to GO. PL emission results from the radiative recombination of photogenerated electrons and holes; therefore, a lower PL intensity indicates a reduced recombination rate and more efficient charge separation (Pritha et al. 2024). The PL spectra of ZnO and ZnO/GO, recorded at an excitation wavelength of 340 nm, are presented in Fig. 6.

As shown in Fig. 6, the ZnO/GO composite exhibited a lower PL intensity than pure ZnO. This reduction can be attributed to the incorporation of GO, which promoted a more effective separation of photogenerated charge carriers. Owing to its excellent electrical conductivity, GO facilitates the rapid transfer of photogenerated electrons from ZnO to the GO structure, thereby suppressing electron–hole recombination (Peng et al. 2025). Similar reductions in charge-carrier recombination have been reported for graphene-based photocatalysts by Bibi et al. (2026), Immanuvel et al. (2025), Le et al. (2024), and Zhang et al. (2025).

Enhanced charge separation is highly desirable for photocatalytic applications because both photogenerated electrons and holes actively participate in the reduction and oxidation reactions responsible for contaminant degradation (Peng et al. 2025).

### Comparative study of the photocatalytic degradation efficiency

**Fig. 7:**
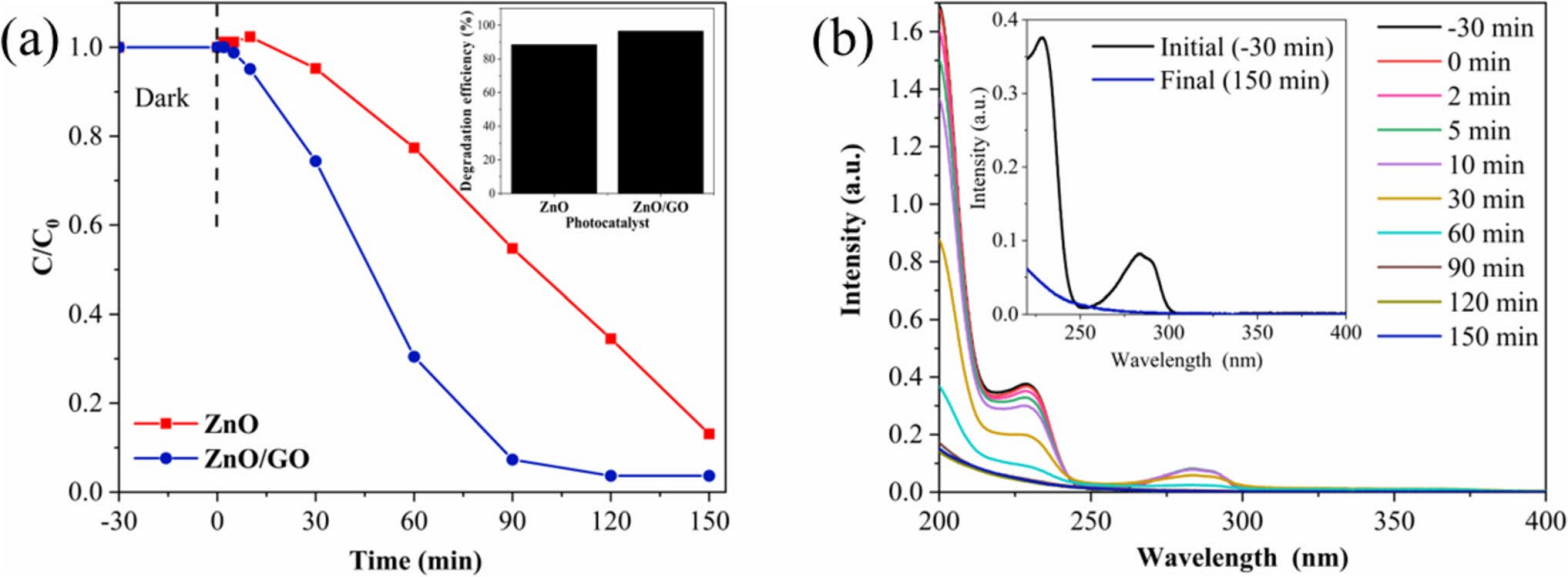
(**a**) Photocatalytic degradation profiles of 2,4-D using ZnO and ZnO/GO; and (**b**) UV–Vis absorption spectra of 2,4-D recorded during the photocatalytic reaction using ZnO/GO, with the inset highlighting the initial and final spectra. Parameters: [2,4-D] = 10 mg L^−1^; [photocatalyst] = 0.25 g L^−1^; pH = 5.0 ± 0.5

Figure 7a presents the photocatalytic degradation profiles of 2,4-D obtained using ZnO and ZnO/GO as photocatalysts, while Fig. 7b shows the UV–Vis absorption spectra recorded during the degradation process of 2,4-D in the presence of the ZnO/GO photocatalyst.

As shown in Fig. 7a, both ZnO and ZnO/GO exhibited low adsorption capacities during the dark adsorption period (−30 to 0 min), resulting in a maximum contaminant removal of approximately 3%. This finding indicates that adsorption played only a minor role in the overall removal process.

Under photocatalytic conditions, ZnO/GO exhibited superior performance compared with pure ZnO in the degradation of the herbicide 2,4-D. After 150 min of irradiation, degradation efficiencies of 96.6% and 88.5% were achieved for ZnO/GO and ZnO, respectively. These results demonstrate that the incorporation of GO significantly enhanced the photocatalytic activity of ZnO.

The improved performance of the ZnO/GO composite can be attributed to several factors. First, the lower electron–hole recombination rate observed in the PL analysis indicates more efficient charge separation. Second, the smaller particle size determined by DLS may have increased the available surface area and the number of active sites. In addition, the enhanced optical absorption observed in the diffuse reflectance spectra favors the utilization of incident light, contributing to the improved photocatalytic efficiency.

The UV–Vis spectra recorded during the degradation process (Fig. 7b) further confirm the effectiveness of the ZnO/GO photocatalyst. The characteristic absorption bands of 2,4-D, located at approximately 230 and 283 nm, completely disappeared after 150 min of photocatalytic treatment. This result indicates the complete degradation of the parent 2,4-D molecule under the investigated conditions.

### Evaluation of the chemical stability of the photocatalysts

**Fig. 8:**
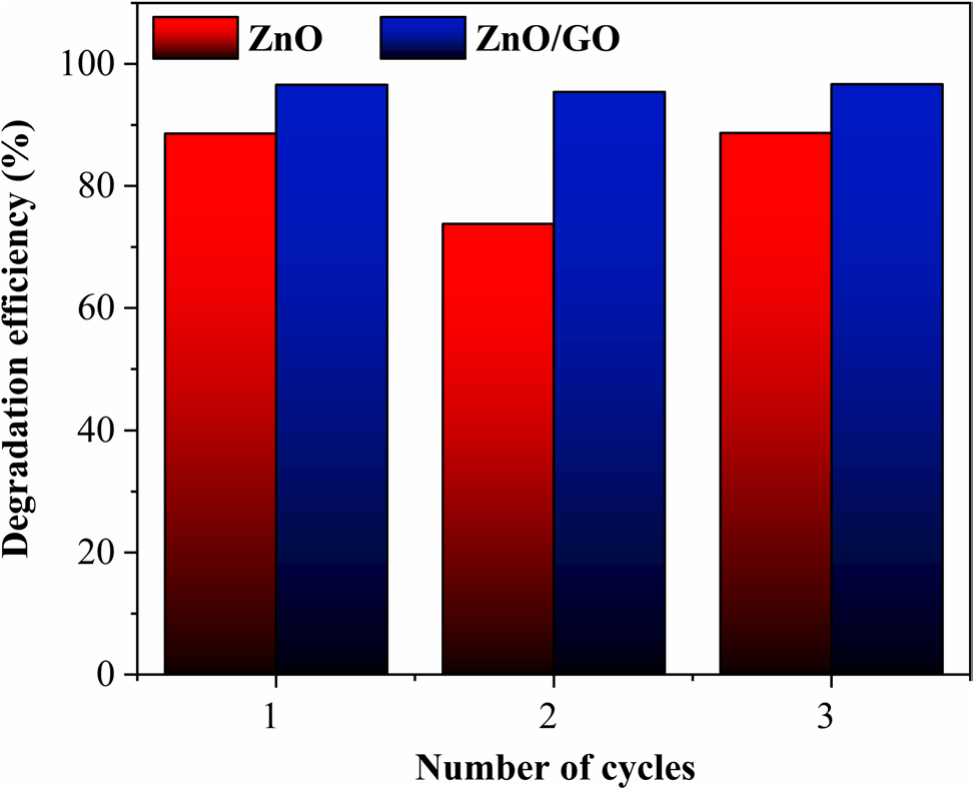
Reuse of ZnO and ZnO/GO photocatalysts over three consecutive cycles. Parameters: [2,4-D] = 10 mg L^−1^; [photocatalyst] = 0.25 g L^−1^; pH = 5.0 ± 0.5; time = 150 min

The chemical stability of the ZnO and ZnO/GO photocatalysts was assessed through reuse experiments conducted under the same operating conditions described previously. Figure 8 presents the degradation efficiencies obtained over three consecutive photocatalytic cycles using ZnO and ZnO/GO.

The chemical stability of a photocatalyst is a critical parameter for assessing its potential applicability in practical environmental remediation processes. As shown in Fig. 8, both ZnO and ZnO/GO maintained their photocatalytic performance over three consecutive reuse cycles, indicating good chemical stability under the investigated conditions.

### Total organic carbon (TOC) analysis

To assess the mineralization of 2,4-D during the photocatalytic process, total organic carbon (TOC) analyses were performed on the initial solution and on the treated solutions obtained after photocatalytic degradation using ZnO and ZnO/GO photocatalysts. The concentrations of total organic carbon (TOC), inorganic carbon (IC), and total carbon (TC), together with the corresponding TOC removal efficiencies, are summarized in Table 2.

**Table 2:** Concentrations of TOC, IC, and TC, and TOC removal efficiency (%) for the initial 2,4-D solution and those obtained after treatment with ZnO or ZnO/GO

2,4-D solution	TOC (mgL^−1^)	IC (mgL^−1^)	TC (mgL^−1^)	TOC Removal (%)
Initial	4.796	0.5428	5.339	*
Treated with ZnO	1.266	1.840	3.106	73.60
Treated with ZnO/GO	0.7282	1.702	2.430	84.82

* Not applicable

The solution treated with the ZnO/GO photocatalyst exhibited a lower TOC concentration than that treated with ZnO, indicating a higher degree of 2,4-D mineralization. This result further confirms the beneficial effect of GO incorporation into the photocatalyst, as the composite promotes photogenerated charge separation and reduces electron–hole recombination, thereby enhancing photocatalytic activity.

### Ecotoxicity assessment using deva lettuce seeds

Exposure of lettuce seeds to 2,4-D may induce phytotoxic effects, as reported by Rebesquini et al. (2024), Salcedo et al. (2021), and Roesler et al. (2020). Therefore, ecotoxicity tests using treated solutions are important for evaluating not only the efficiency of contaminant degradation but also the potential residual toxicity resulting from degradation byproducts. Comparisons of the germination index (GI) and relative germination index (RGI) obtained for seeds exposed to untreated and treated solutions may provide evidence of reduced phytotoxicity following photocatalytic treatment and indicate the absence of significant residual toxic effects on the tested organism.

**Fig. 9:**
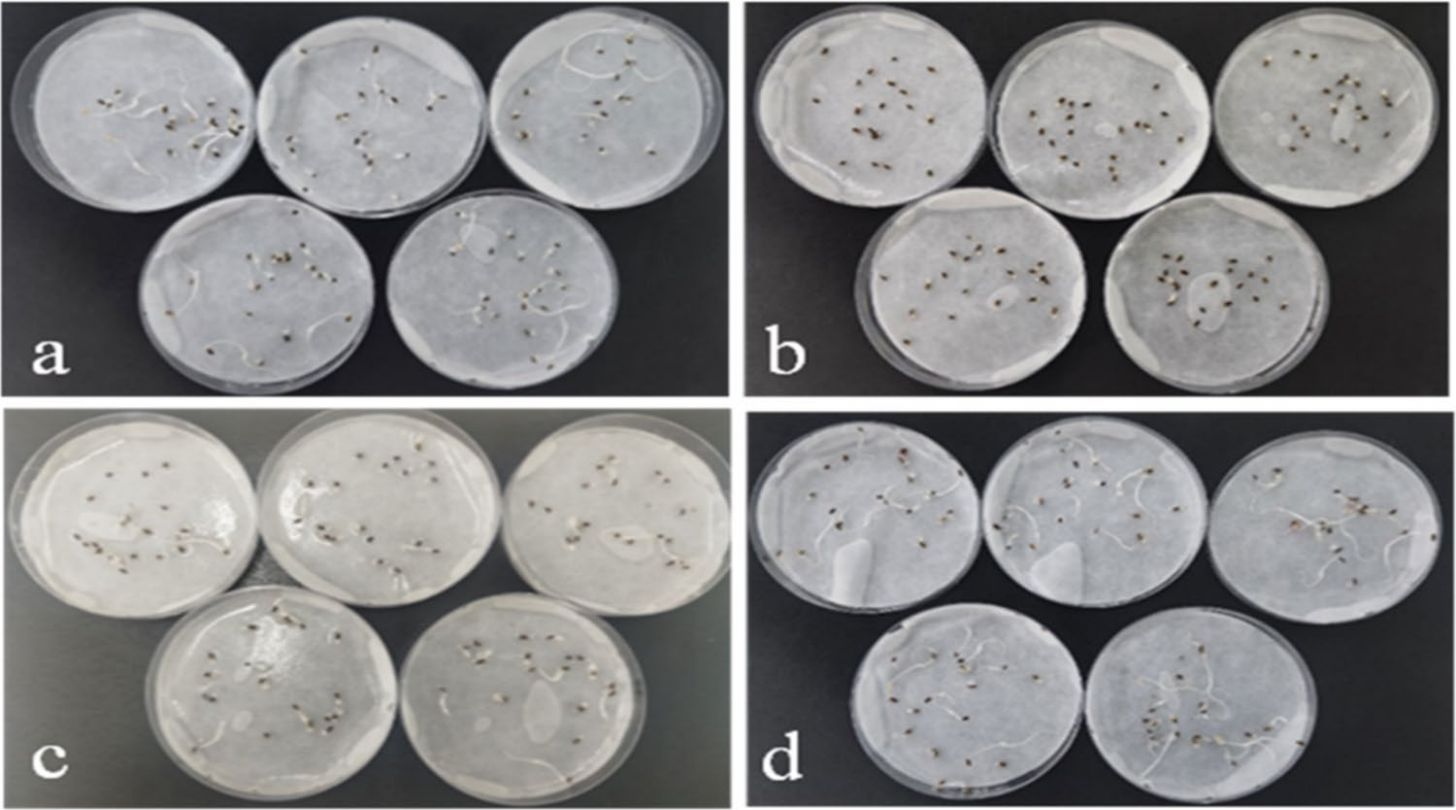
Petri dishes containing Deva lettuce seeds after incubation at 22 ± 2 °C for 120 h: (**a**) negative control (NC); (**b**) untreated 2,4-D solution; and 2,4-D solutions after photocatalytic treatment using (**c**) ZnO and (**d**) ZnO/GO

The experiments were conducted in quintuplicate, as described previously. Deva lettuce seeds were exposed to untreated 2,4-D solutions and to solutions treated by heterogeneous photocatalysis using ZnO or ZnO/GO as photocatalysts. Figure 9 shows representative Petri dishes containing Deva lettuce seeds after incubation at 22 ± 2 °C for 120 h.

As seen in Fig. 9b, exposure to the untreated 2,4-D solution resulted in low seed germination and limited root development. In contrast, seeds exposed to the solution treated with ZnO/GO exhibited germination and root growth comparable to those observed for the negative control. These observations suggest a substantial reduction in the phytotoxicity of the solution following photocatalytic treatment with the ZnO/GO composite. The ecotoxicity parameters determined for Deva lettuce seeds (*Lactuca sativa*) are presented in Table 3.

**Table 3:** Ecotoxicity parameters with Deva lettuce seeds 854-*Lactuca Sativa* (Isla Sementes Ltda; Germination: 97%; Purity: 100%; Lot 172,216–001)

Solution	GI (%)	RGI	Classification by Soares et al. (2013)
Negative control (NC)	98 ± 2.45	*	*
Initial 2,4-D	45 ± 19.74	0.0763 ± 0.021	Phytotoxic
Treated with ZnO	93 ± 4.00	0.2747 ± 0.068	Non-phytotoxic
Treated with ZnO/GO	96 ± 3.74	0.8914 ± 0.021	Non-phytotoxic

* Not applicable

The GI values obtained in the ecotoxicity tests were interpreted according to the classification scale proposed by Soares et al. (2013). The untreated 2,4-D solution was classified as phytotoxic, presenting GI values between 40 and 60%. In contrast, the solutions treated by photocatalysis using ZnO or ZnO/GO were classified as non-phytotoxic, with GI values ranging from 80 to 100%. These results indicate that heterogeneous photocatalysis using either ZnO or ZnO/GO effectively reduced the phytotoxicity of the herbicide solution for this species.

Furthermore, the relative root growth index (RGI) of seeds exposed to the solution treated with ZnO/GO was 3.2 times higher than that obtained for the solution treated with ZnO, indicating a greater reduction in residual phytotoxicity when the composite photocatalyst was employed. It is important to note that no seed germination was observed in the positive control; therefore, its corresponding values are not presented in Table 3.

### Comparative study of photolysis, adsorption, and photocatalysis

**Fig. 10:**
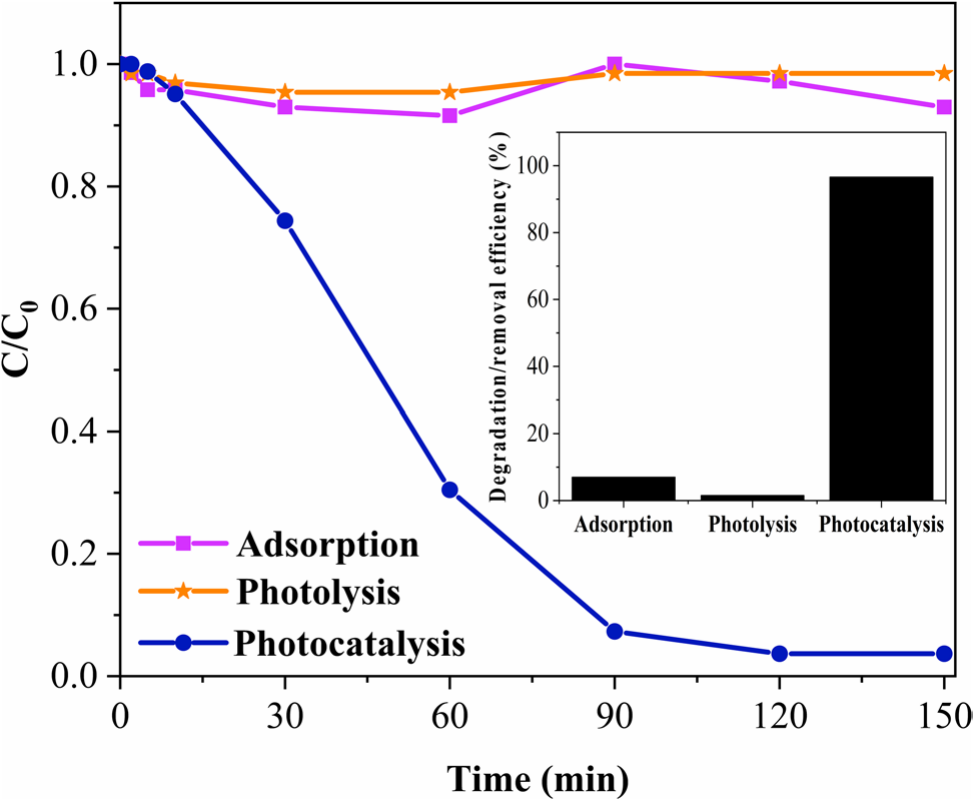
Degradation/removal profiles of 2,4-D by adsorption, photolysis, and photocatalysis. Parameters: [2,4-D] = 10 mg L^−1^; [ZnO/GO] = 0.25 g L^−1^; pH = 5.0 ± 0.5; time = 150 min

Figure 10 presents the degradation/removal profiles of 2,4-D obtained through photolysis, adsorption, and photocatalysis. The ZnO/GO sample was selected for the adsorption and photocatalysis experiments because it exhibited the highest photocatalytic performance in the preliminary studies described above.

After 150 min, adsorption accounted for only 7% of herbicide removal. This limited performance may be associated with the relatively low surface area of the photocatalyst. Under photolysis, approximately 1.5% of the 2,4-D was degraded, whereas photocatalysis achieved 96.6% degradation. These results clearly demonstrate that photocatalysis was the predominant mechanism responsible for 2,4-D degradation under the investigated conditions.

### Effect of catalyst dosage

**Fig. 11:**
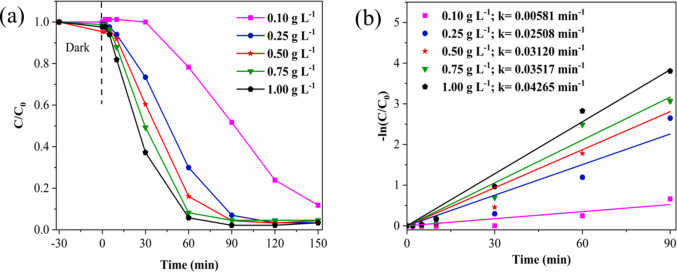
(**a**) Photocatalytic degradation profiles and (**b**) pseudo-first-order kinetic plots for 2,4-D degradation at different photocatalyst dosages. Parameters: [2,4-D] = 10 mg L^−1^; pH = 5.0 ± 0.5; time = 150 min

Figure 11a shows the photocatalytic degradation profiles of 2,4-D obtained at different catalyst dosages, while Fig. 11b presents the corresponding pseudo-first-order kinetic fits. The catalyst concentration was varied from 0.1 to 1.0 g L^−1^ while maintaining an initial 2,4-D concentration of 10 mg L^−1^ and pH 5.0 ± 0.5. Increasing the catalyst dosage from 0.1 to 1.0 g L^−1^ resulted in a progressive enhancement of the photodegradation rate, with pseudo-first-order kinetic constants ranging from 0.00581 to 0.04265 min^−1^.

Within the investigated range (0.1–1.0 g L^−1^), no detrimental effect associated with increasing catalyst dosage was observed. On the contrary, the pseudo-first-order kinetic constant increased by a factor of 7.34 when the catalyst concentration was raised from 0.1 to 1.0 g L^−1^. This improvement can be attributed to the increase in available surface area and active sites, which enhances the generation of charge carriers and reactive oxygen species, such as hydroxyl radicals (^•^OH) and superoxide radicals (O_2_^•−^), thereby promoting pollutant degradation (Farooq et al. 2022).

In photocatalytic systems where adsorption plays an important role, increasing the catalyst dosage generally enhances degradation due to the greater availability of adsorption sites. However, the adsorption experiments performed in this study demonstrated that 2,4-D exhibited negligible adsorption onto the catalyst surface. Therefore, it is likely that dissolved H₂O and O₂ interact with the active sites of the photocatalyst, generating reactive radicals that drive the degradation process.

It is worth noting that excessive catalyst dosages may eventually reduce photocatalytic performance. Two main factors have been proposed to explain this behavior. First, increased suspension turbidity can promote light scattering and reduce photon penetration into the solution, decreasing the amount of light available for photocatalytic reactions. Second, catalyst concentrations above the optimum value may favor particle agglomeration, reducing the effective surface area, promoting sedimentation, and decreasing the number of active sites available for light absorption and photocatalytic reactions (Farooq et al. 2022; Rahman et al. 2024; Esfandian et al. 2024; Adnan et al. 2025).

### Effect of initial herbicide (2,4-D) concentration

**Fig. 12:**
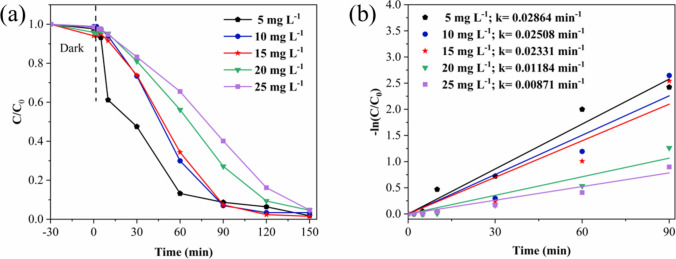
(**a**) Photocatalytic degradation profiles and (**b**) pseudo-first-order kinetic plots for 2,4-D degradation at different initial herbicide concentrations. Parameters: [ZnO/GO] = 0.25 g L^−1^; time = 150 min; and natural pH of the solution

The photocatalytic degradation profiles obtained by varying the initial 2,4-D concentration from 5 to 25 mg L^−1^ are presented in Fig. 12a, while the corresponding pseudo-first-order kinetic fits are shown in Fig. 12b.

The highest degradation efficiency (98.1%) was achieved at an initial 2,4-D concentration of 5 mg L^−1^. As the herbicide concentration increased from 5 to 25 mg L^−1^, the degradation efficiency decreased from 98.1 to 95.3%. A corresponding reduction in the photodegradation rate was also observed, with the kinetic constant decreasing from 0.02864 m^−1^ at 5 mg L^−1^ to 0.00871 min^−1^ at 25 mg L^−1^.

The decrease in photocatalytic performance at higher herbicide concentrations may be attributed to the occupation of active sites by 2,4-D molecules and to competition for incident light between the pollutant molecules and the photocatalyst (Immanuvel et al. 2025). Furthermore, although the concentration of 2,4-D increased, the amount of reactive species generated by the photocatalyst, including electron–hole pairs, ^•^OH radicals, and O_2_^•−^ radicals, remained unchanged. As a result, the available reactive species became insufficient to efficiently react with the larger number of herbicide molecules present in solution (Ding et al. 2019; Huong et al. 2024).

### Effect of reactive species scavengers on the photocatalytic degradation of 2,4-D

**Fig. 13:**
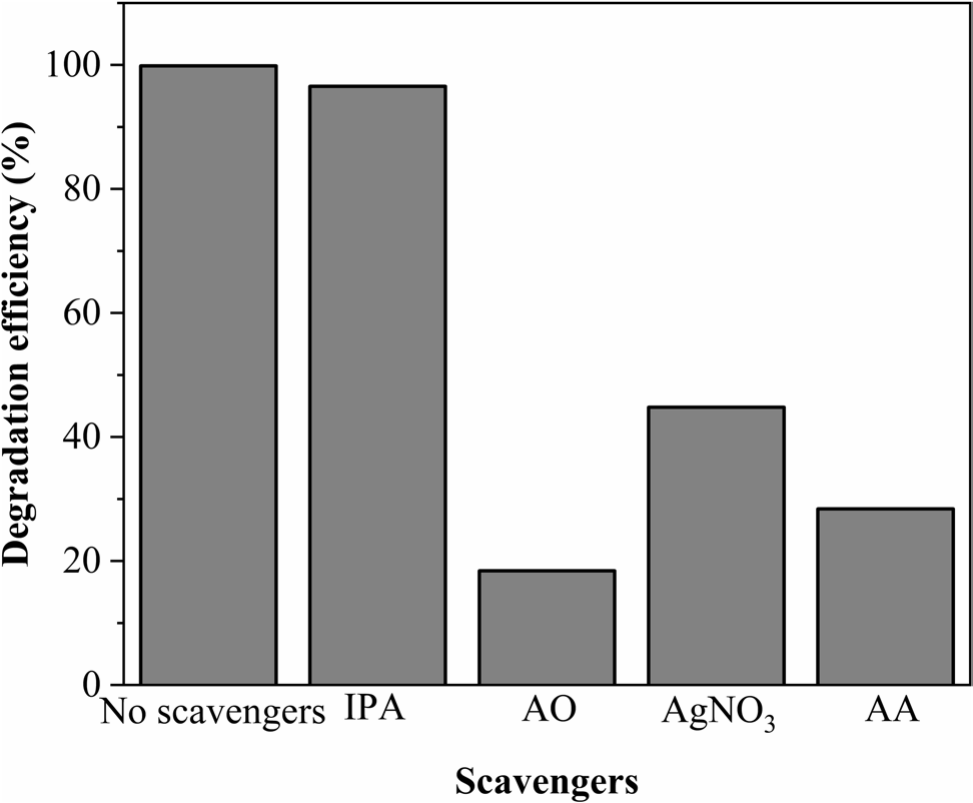
Degradation efficiency of 2,4-D in the presence of reactive species scavengers. Parameters: [2,4-D] = 10 mg L^−1^; [ZnO/GO] = 0.25 g L^−1^; time = 150 min

The results presented in Fig. 13 revealed that the relative importance of the investigated reactive species in the degradation of 2,4-D followed the order: h^+^  > O_2_^•−^ > e^−^ > ^•^OH. In the presence of the corresponding scavengers, the degradation efficiencies decreased to 18.43% (h^+^), 28.4% (O_2_^•−^), 44.81% (e^−^), and 96.56% (^•^OH). These findings indicate that photogenerated holes and superoxide radicals are the predominant reactive species involved in the degradation process, as their suppression resulted in a substantial reduction in photocatalytic efficiency.

The observed behavior is consistent with previous studies reported in the literature, which have highlighted the crucial role of superoxide radicals (O_2_^•−^) in the photocatalytic degradation of 2,4-D (Kumar et al. 2019; Pattappan et al. 2023; Lan et al. 2024). Therefore, the scavenger experiments provided important mechanistic evidence for elucidating the photocatalytic degradation pathway of 2,4-D using the ZnO/GO composite.

### Proposed mechanism for the photocatalytic degradation of 2,4-D using the ZnO/GO composite

The photocatalytic degradation mechanism of 2,4-dichlorophenoxyacetic acid (2,4-D) using the ZnO/GO composite was proposed based on the results obtained from the radical-scavenging experiments and is illustrated by Eqs. 8–22.

Photocatalyst activation:

8 $$\mathrm{Z}\mathrm{n}\mathrm{O}/\mathrm{G}\mathrm{O}+\mathrm{h}\upnu \to \mathrm{Z}\mathrm{n}\mathrm{O}({\mathrm{e}}^{-};{\mathrm{h}}^{+})/\mathrm{G}\mathrm{O}$$

Electron transfer from ZnO to GO:

9 $$\mathrm{Z}\mathrm{n}\mathrm{O}{(\mathrm{e}}^{-};{\mathrm{h}}^{+})/\mathrm{G}\mathrm{O}\to \mathrm{Z}\mathrm{n}\mathrm{O}({\mathrm{h}}^{+})/\mathrm{G}\mathrm{O}{(\mathrm{e}}^{-})$$

Adsorption of 2,4-D:

10 $$\mathrm{Z}\mathrm{n}\mathrm{O}\left(\mathrm{h}^+\right)/\mathrm{G}\mathrm{O}(\mathrm{e}^-)+\mathrm{2,4}-\mathrm{D}\rightarrow\mathrm{Z}\mathrm{n}\mathrm{O}\left(\mathrm{h}^+\right).\mathrm{2,4}-\mathrm{D}/\mathrm{G}\mathrm{O}(\mathrm{e}^-)$$

Adsorption of dissolved oxygen:

11 $$\mathrm{Z}\mathrm{n}\mathrm{O}\left(\mathrm{h}^+\right).\mathrm{2,4}-\mathrm{D}/\mathrm{G}\mathrm{O}(\mathrm{e}^-)+{\mathrm{O}}_2\rightarrow\mathrm{Z}\mathrm{n}\mathrm{O}\left(\mathrm{h}^+\right).\mathrm{2,4}-\mathrm{D}/\mathrm{G}\mathrm{O}(\mathrm{e}^-).{\mathrm{O}}_2$$

Formation of superoxide radicals (or reactive intermediates):

12 $$\mathrm{Z}\mathrm{n}\mathrm{O}\left(\mathrm{h}^+\right).\mathrm{2,4}-\mathrm{D}/\mathrm{G}\mathrm{O}(\mathrm{e}^-).\;{\mathrm{O}}_2\rightarrow\mathrm{O}_2^{\bullet-}+\mathrm{Z}\mathrm{n}\mathrm{O}\left(\mathrm{h}^+\right).\;\mathrm{2,4}-\mathrm{D}/\mathrm{G}\mathrm{O}$$

Initiation of 2,4-D degradation via reduction:

13 $$\mathrm{O}_2^{\bullet-}+\mathrm{Z}\mathrm{n}\mathrm{O}\left(\mathrm{h}^+\right).\mathrm{2,4}-\mathrm{D}/\mathrm{G}\mathrm{O}\rightarrow(\mathrm{2,4}-{\mathrm{D})}^{\bullet-}+\mathrm{Z}\mathrm{n}\mathrm{O}\left(\mathrm{h}^+\right)/\mathrm{G}\mathrm{O}+{\mathrm{O}}_2$$

14 $$\mathrm{Z}\mathrm{n}\mathrm{O}\left(\mathrm{h}^+\right).\;\mathrm{2,4}-\mathrm{D}/\mathrm{G}\mathrm{O}\left(\mathrm{e}^-\right)\rightarrow(\mathrm{2,4}-{\mathrm{D})}^{\bullet-}+\mathrm{Z}\mathrm{n}\mathrm{O}\left(\mathrm{h}^+\right)/\mathrm{G}\mathrm{O}$$

Photocatalyst regeneration:

15 $$\mathrm{Z}\mathrm{n}\mathrm{O}\left(\mathrm{h}^+\right)/\mathrm{O}\mathrm{G}+{\mathrm{H}}_2\mathrm{O}\rightarrow\mathrm{Z}\mathrm{n}\mathrm{O}/\mathrm{O}\mathrm{G}+{\mathrm{O}\mathrm{H}}^\bullet+\mathrm H^+$$

Formation of superoxide radicals from hydroxyl radicals:

16 $${\mathrm{O}\mathrm{H}}^\bullet+{\mathrm{O}\mathrm{H}}^\bullet\rightarrow{\mathrm{H}}_2{\mathrm{O}}_2$$

17 $${\mathrm{H}}_2{\mathrm{O}}_2+{\mathrm{O}\mathrm{H}}^\bullet\rightarrow\mathrm{HO}_2^\bullet+{\mathrm{H}}_2\mathrm{O}$$

18 $$\mathrm{HO}_2^\bullet\rightleftharpoons{\mathrm{O}_2^{\bullet-}+\mathrm{H}}^+$$

Degradation of 2,4-D:

19 $$\mathrm{O}_2^{\bullet-}+\mathrm{2,4}-\mathrm{D}\rightarrow(\mathrm{2,4}-{\mathrm{D})}^{\bullet-}$$

20 $$(\mathrm{2,4}-{\mathrm{D})}^{\bullet-}+{\mathrm{H}}_2\mathrm{O}\rightarrow\mathrm{I}\mathrm{n}\mathrm{t}\mathrm{e}\mathrm{r}\mathrm{m}\mathrm{e}\mathrm{d}\mathrm{i}\mathrm{a}\mathrm{t}\mathrm{e}\mathrm{s}$$

21 $$(\mathrm{2,4}-{\mathrm{D})}^{\bullet-}+{\mathrm{O}}_2\rightarrow\mathrm{I}\mathrm{n}\mathrm{t}\mathrm{e}\mathrm{r}\mathrm{m}\mathrm{e}\mathrm{d}\mathrm{i}\mathrm{a}\mathrm{t}\mathrm{e}\mathrm{s}$$

Mineralization:

22 $$\mathrm{I}\mathrm{n}\mathrm{t}\mathrm{e}\mathrm{r}\mathrm{m}\mathrm{e}\mathrm{d}\mathrm{i}\mathrm{a}\mathrm{t}\mathrm{e}\mathrm{s}\to {\mathrm{H}}_{2}\mathrm{O}+{\mathrm{C}\mathrm{O}}_{2}+\mathrm{H}\mathrm{C}\mathrm{l}$$

The photocatalytic degradation mechanism of 2,4-dichlorophenoxyacetic acid (2,4-D) over the ZnO/GO composite was proposed based on the results obtained from the radical-scavenging experiments and is illustrated by Eqs. 8–22.

Upon irradiation, the photocatalyst is activated by the absorption of photons with energy equal to or greater than its band-gap energy, leading to the generation of photogenerated electron–hole pairs (e^−^/h^+^) (Eq. 8). The photogenerated electrons are subsequently transferred to the GO structure, where they are effectively trapped, thereby reducing electron–hole recombination (Eq. 9). Graphene-based materials, such as GO, are well known for acting as electron acceptors, thereby delaying charge-carrier recombination and enhancing photocatalytic performance (Hernández-Del Castillo et al. 2022).

The positively charged holes in the ZnO structure serve as active sites for the adsorption of 2,4-D molecules. Under the experimental conditions employed in this study, 2,4-D is predominantly present in its anionic form and can be attracted to these positively charged sites through electrostatic interactions (Eq. 10). Dissolved oxygen is adsorbed onto the photocatalyst surface (Eq. 11) and subsequently captures the photogenerated electrons, yielding superoxide radicals (O_2_^•−^) (Eq. 12). These adsorbed superoxide radicals may act as reducing agents toward the herbicide, generating the 2,4-D radical anion (2,4-D^•−^) (Eq. 13).

Alternatively, electrons trapped within the GO structure may be directly transferred to the adsorbed 2,4-D molecules, also leading to the formation of 2,4-D^•−^ (Eq. 14). Water molecules adsorbed on the catalyst surface are oxidized by photogenerated holes, producing hydroxyl radicals (^•^OH) (Eq. 15). According to the scavenger experiments, hydroxyl radicals do not appear to play a major direct role in the degradation of 2,4-D. However, they may participate indirectly by promoting a series of reactions that generate additional superoxide radicals in the reaction medium (Eqs. 16–18).

The superoxide radicals generated in solution subsequently react with 2,4-D, producing the radical anion intermediate (2,4-D^•⁻^), which undergoes successive reactions with oxygen and water, yielding a variety of degradation intermediates (Eqs. 19–21). These sequential oxidation and reduction reactions ultimately result in extensive mineralization of the herbicide (Eq. 22), as evidenced by the high total organic carbon (TOC) removal efficiency of 84.82% reported in Table 2.

The proposed mechanism is consistent with previous findings reported by Pattappan et al. (2023), who identified superoxide radicals as the primary reactive species responsible for the degradation of 2,4-D. Using liquid chromatography–mass spectrometry (LC–MS), those authors demonstrated that O_2_^•−^ radicals can attack the C–O bond of the aromatic structure of 2,4-D, producing 2,4-dichlorophenol and glycolic acid as initial degradation products. Subsequently, 2,4-dichlorophenol undergoes dechlorination to form 2-chlorohydroquinone, followed by the formation of hydroxyquinol. Further oxidation and ring-opening reactions of hydroxyquinol generate aliphatic acids, such as maleic and formic acids. Finally, these low-molecular-weight organic acids react with superoxide radicals and are progressively mineralized into CO_2_ and H_2_O.

**Fig. 14:**
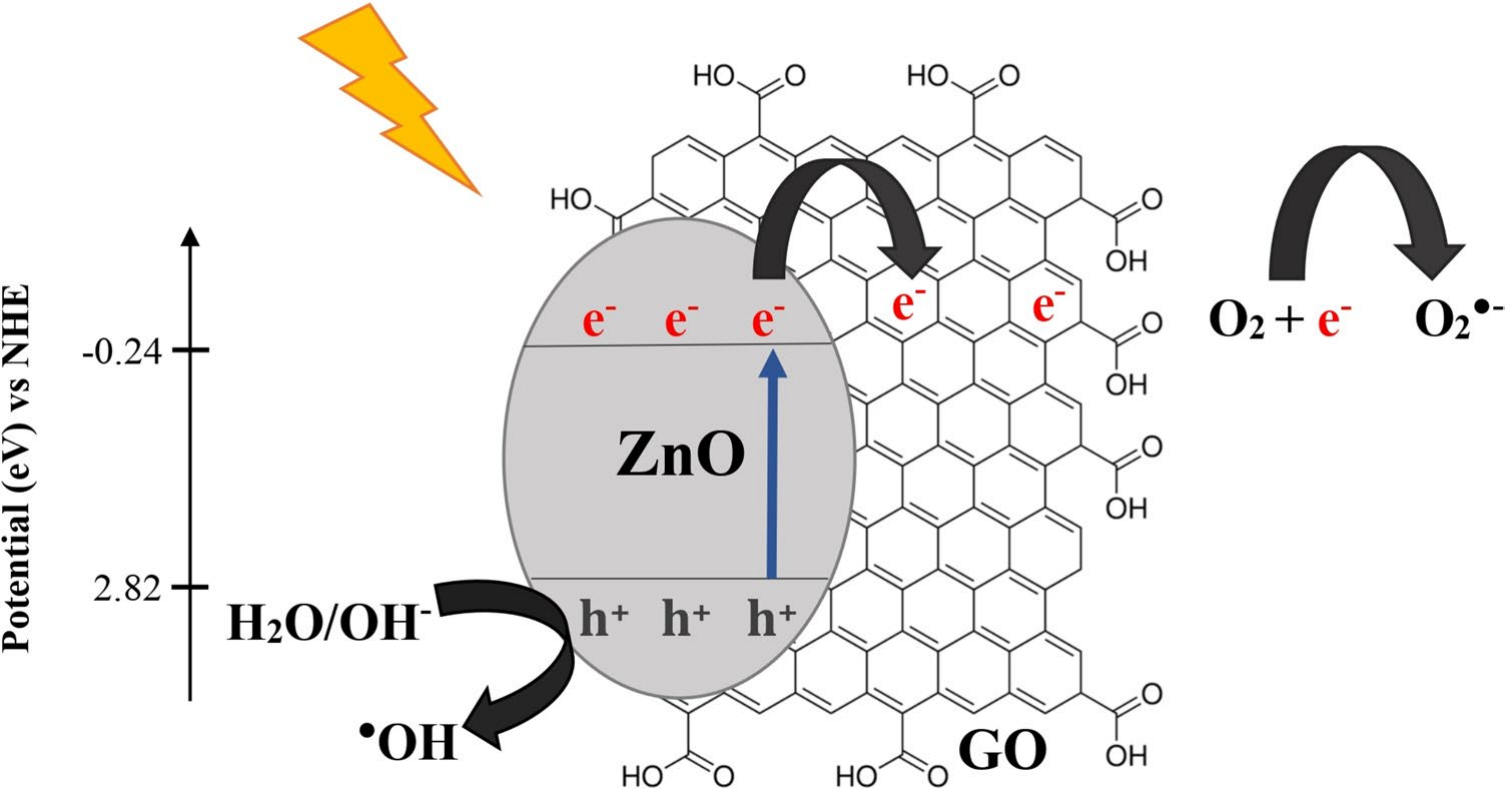
Schematic representation of the proposed electron transfer from ZnO to GO, reducing electron–hole recombination

Figure 14 presents a schematic diagram illustrating the proposed electron-transfer pathway from ZnO to the GO lattice in the ZnO/GO composite. This mechanism was proposed based on the SEM micrograph of the nanocomposite (Fig. 4c), which suggests that the stacked GO sheets act as a support for ZnO particle deposition. The positions of the valence band and conduction band used in the diagram were estimated from Eqs. (6) and (7).

The positions of the conduction band (CB) and valence band (VB) are essential for understanding the redox reactions involved in the photocatalytic process. For the formation of superoxide radicals (O_2_^•⁻^), the conduction-band potential of the photocatalyst must be more negative than the oxygen reduction potential. The standard redox potential of the O_2_/O_2_^•⁻^ couple is approximately − 0.33 V (Bissenova et al. 2025). The superoxide radical is one of the main reactive oxygen species (ROS) involved in photocatalytic processes. Its importance lies not only in its oxidizing ability but also in its role as an intermediate in the formation of other highly reactive species, such as H_2_O_2_ and ^•^OH. Likewise, the valence-band potential must be more positive than 2.34 V [H_2_O/^•^OH] or 1.99 V [OH^−^/^•^OH] to enable the formation of hydroxyl radicals through the oxidation of H_2_O or OH^−^, respectively (Zhang et al. 2019; Li et al. 2021).

Based on the estimated band positions, the direct reduction of O_2_ to O_2_^•⁻^ by photogenerated electrons in pure ZnO is thermodynamically unfavorable because the conduction-band potential of ZnO (−0.24 V) is less negative than the redox potential of the O_2_/O_2_^•⁻^ couple (−0.33 V). However, in the ZnO/GO composite, the photogenerated electrons may be transferred from ZnO to the GO lattice, where they can be temporarily trapped and subsequently participate in reduction reactions involving dissolved oxygen. This process may facilitate the formation of O_2_^•⁻^ radicals, which can initiate a sequence of reactions that contribute to contaminant degradation.

In addition, the transfer of electrons from ZnO to GO may prolong the lifetime of photogenerated holes in the valence band by reducing electron–hole recombination. As a result, these holes remain available for oxidation reactions involving H_2_O, OH^−^, and 2,4-D molecules. Therefore, the incorporation of GO into ZnO is proposed to enhance photocatalytic activity by promoting charge separation, facilitating electron mobility, and increasing the availability of reactive species involved in pollutant degradation. The improved photocatalytic performance of the ZnO/GO composite may thus be attributed to the ability of GO to act as an electron acceptor and transport medium, extending the lifetime of photogenerated charge carriers after photoactivation.

## Conclusions

Both ZnO and the ZnO/GO composite proved to be promising photocatalysts for the degradation of the herbicide 2,4-D through heterogeneous photocatalysis. High degradation efficiencies were achieved, reaching 88.5% for ZnO and 96.6% for ZnO/GO after 150 min of reaction. Likewise, significant mineralization was observed, with TOC removal efficiencies of 73.60% and 84.82% for ZnO and ZnO/GO, respectively. Both materials also demonstrated good chemical stability over three consecutive reuse cycles.

Ecotoxicity assays further showed that the treated solutions were non-phytotoxic to Deva lettuce seeds (*Lactuca sativa*), indicating that the photocatalytic treatment effectively reduced the toxicity associated with the herbicide.

Among the evaluated materials, the ZnO/GO composite exhibited superior performance in all investigated parameters, demonstrating that the incorporation of GO enhanced the photocatalytic activity of ZnO. This improvement is likely associated with the combined effects of:

- The role of GO as a support for ZnO deposition, promoting a more homogeneous dispersion of ZnO particles and reducing agglomeration;
- Enhanced optical absorption in the visible-light region, reflected by the reduction in the band-gap energy of the composite;
- Lower photoluminescence intensity, indicating reduced electron–hole recombination and more efficient charge separation, which favors the oxidation and reduction reactions occurring on the photocatalyst surface.

Overall, the results demonstrate that GO improved the photocatalytic performance of ZnO, making the ZnO/GO composite a promising material for the treatment of water contaminated with herbicides such as 2,4-D. These findings highlight the potential of ZnO/GO nanocomposites as efficient, stable, and environmentally compatible photocatalysts for the remediation of water contaminated by emerging agrochemical pollutants.

## Data Availability

The authors confirm that the data supporting the findings of this study are available within the article.
